# Supplementary material for: Patient Attitudes Toward Artificial Intelligence in Cancer Care: Scoping Review
Source: JMIR Cancer. 2025 Aug 22;11:e74010. doi: 10.2196/74010 (PMC12373359; doi:10.2196/74010)
Supplement: Multimedia Appendix 1 [file cancer-v11-e74010-s001.pdf]

Search Terms

MEDLINE (OVID)	
**Cancer**	(cancer* or neoplas* or malignan* or tumor*).tw,kw,kf.
	OR
	exp Neoplasms/
AND	
**Artificial Intelligence**	(AI OR "artificial intelligence" or "computational intelligence" or "computer reasoning" or "computer vision systems" or ("knowledge Acquisition" adj2 computer*) or ("knowledge representation" adj2 computer*) or "Machine Intelligence" or "machine learning" or "natural language processing" or "computer neural network*" or "computational neural network*" or "connectionist model*" or "models neural network*" or "neural networks*" or perceptron* or "deep learning" or "hierarchical learning" or "data mining" or "text mining").tw,kw,kf.
	OR
	exp "Artificial Intelligence"/ or exp "Data Mining"/
AND	
**Patients' Perspective**	(patient* adj4 ("critical thinking" OR perception* OR perspective* OR emotion* OR feeling* OR regret* OR attitude* OR opinion* OR sentiment* OR "mental process*" OR trust OR distrust OR belief* OR experience*)).tw,kw,kf.
	OR
	Attitude/ or exp Emotions/
**EMBASE (OVID)**	
**Cancer**	(cancer* or neoplas* or malignan* or tumor*).ab,kf,ti.
	OR
	exp Neoplasms/
AND	
**Artificial Intelligence**	(AI OR "artificial intelligence" or "computational intelligence" or "computer reasoning" or "computer vision systems" or ("knowledge Acquisition" adj2 computer*) or ("knowledge representation" adj2 computer*) or "Machine Intelligence" or "machine learning" or "natural language processing" or computer neural network* or computational neural network* or connectionist model* or models neural network* or neural networks* or perceptron* or "deep learning" or "hierarchical learning" or "data mining" or "text mining").ab,kf,ti.
	OR
	exp "Artificial Intelligence"/ or exp "Data Mining"/

AND	
**Patients' Perspective**	(patient* adj4 ("critical thinking" OR perception* OR perspective* OR emotion* OR feeling* OR regret* OR attitude* OR opinion* OR sentiment* OR "mental process*" OR trust OR distrust OR belief* OR experience*)).ab,kf,ti.
	OR
	Attitude/ or exp Emotion/
**PsychInfo (EBSCO)**	
**Cancer**	cancer* or neoplas* or malignan* or tumor*
	OR
	DE "Neoplasms" OR DE "Benign Neoplasms" OR DE "Breast Neoplasms" OR DE "Childhood Neoplasms" OR DE "Digestive System Neoplasms" OR DE "Endocrine Neoplasms" OR DE "Leukemias" OR DE "Lung Neoplasms" OR DE "Metastasis" OR DE "Nervous System Neoplasms" OR DE "Skin Neoplasms" OR DE "Terminal Cancer"
AND	
**Artificial Intelligence**	"AI" or "artificial intelligence" or "computational intelligence" or "computer reasoning" or "computer vision systems" or ("knowledge Acquisition" N2 computer*) or ("knowledge representation" N2 computer*) or "machine intelligence" or "machine learning" or "natural language processing" or "computer neural network*" or "computational neural network*" or "connectionist model*" or "models neural network*" or "neural networks*" or perceptron* or "deep learning" or "hierarchical learning" or "data mining" or "text mining"
	OR
	DE "Data Mining" OR DE "Text Analysis" OR DE "Artificial Intelligence" OR DE "Affective Computing" OR DE "Artificial Intelligence Ethics" OR DE "Cognitive Computing" OR DE "Computer Assisted Diagnosis" OR DE "Computer Linguistics" OR DE "Computer Vision" OR DE "Expert Systems" OR DE "Fuzzy Logic" OR DE "Heuristics" OR DE "Intelligent Agents" OR DE "Knowledge Representation" OR DE "Machine Learning" OR DE "Robotics"
AND	
**Patients' Perspective**	patient* N4 ("critical thinking" or perception* or perspective* or emotion* or feeling* or regret* or attitude* or opinion* or sentiment* or "mental process*" or trust or distrust or belief* or experience*)
	OR
	DE "Attitudes" OR DE "Emotions" OR DE "Affective Valence" OR DE "Emotional Content" OR DE "Emotional Health" OR DE "Emotional Intelligence" OR DE "Emotional Processing" OR DE "Emotional Regulation" OR DE "Emotional Responses" OR DE "Emotional States" OR DE "Emotional Style" OR DE "Emotional Support" OR DE "Expressed Emotion"

CINAHL (EBSCO)	
**Cancer**	cancer* or neoplas* or malignan* or tumor*
	OR
	MH "Neoplasms+"
AND	
**Artificial Intelligence**	"AI" or "artificial intelligence" or "computational intelligence" or "computer reasoning" or "computer vision systems" or ("knowledge Acquisition" N2 computer*) or ("knowledge representation" N2 computer*) or "Machine Intelligence" or "machine learning" or "natural language processing" or "computer neural network*" or "computational neural network*" or "connectionist model*" or "models neural network*" or "neural networks*" or perceptron* or "deep learning" or "hierarchical learning" or "data mining" or "text mining"
	OR
	MH "Data Mining+" OR MH "Artificial Intelligence+"
**Patients' Perspective**	patient* N4 ("critical thinking" or perception* or perspective* or emotion* or feeling* or regret* or attitude* or opinion* or sentiment* or "mental process*" or trust or distrust or belief* or experience*)
	OR
	MH "Attitude" OR MH "Emotions+"
