# Supplementary material for: Patient Attitudes Toward Artificial Intelligence in Cancer Care: Scoping Review
Source: JMIR Cancer. 2025 Aug 22;11:e74010. doi: 10.2196/74010 (PMC12373359; doi:10.2196/74010)
Supplement: Multimedia Appendix 3 [file cancer-v11-e74010-s003.pdf]

Studies Included in the Scoping Review by Cancer Type and Patient Count

Cancer Type	No. of Studies [a]	No. of Patients [b]
Breast	6	263
Colorectal	6	251
Esophageal	1	26
Gastric	2	25
Gynecological	4	60
Liver	2	39
Lung	2	135
Lymphoma	1	36
Melanoma	3	375
Meningioma	1	18
Pancreatic	1	17
Pharyngeal	1	40
Prostate	4	323
Soft tissue sarcoma	1	25
Thyroid	1	11
Other	5	63
Unknown	5	421

[a]Studies that include populations with multiple cancer types are listed under each cancer type for which they report findings.
[b]Includes data from one study[18] that included multiple tumor types for some patients.
